# Cognitive Trajectory of COVID-19 and Long COVID in Adult Survivors

**DOI:** 10.1192/j.eurpsy.2022.363

**Published:** 2022-09-01

**Authors:** K. Vakani, M. Ratto, A. Sandford-James, E. Antonova, V. Kumari

**Affiliations:** 1 Brunel University London, Centre For Cognitive Neuroscience, London, United Kingdom; 2 Beingwell, Thinkingwell, Sheffield, United Kingdom

**Keywords:** cognitive function, Covid-19, Long COVID

## Abstract

**Introduction:**

Cognitive functioning and psychological well-being are considered negatively affected by COVID-19. An estimated 15%-40% of COVID-19 patients report disrupted cognitive performance. Higher rates of anxiety, depression and sleep disturbances are also reported post infection.

**Objectives:**

We examined the profile of cognitive changes in a group of adults with a confirmed COVID-19 diagnosis, compared to those without a COVID-19 diagnosis (cross-sectional between-subjects investigation); and for a subgroup, compared to their pre-COVID-19 cognitive function (longitudinal within-subjects investigation).

**Methods:**

One hundred and twenty-one adults (57 with no known history of COVID-19; 64 with confirmed COVID-19; 17/64 with long COVID symptoms) were assessed online for psychological well-being and cognitive function (attention, processing speed, working memory, episodic memory and executive function). Pre-COVID-19 cognitive data were available for 56 of 121 adults (24 adults with a confirmed diagnosis of COVID-19; 22 with no known history of COVID-19) through the MyCognition database.

**Results:**

The COVID-19 group showed reduced processing speed in both cross-sectional and longitudinal investigations, and also showed significant attentional impairment when examined cross-sectionally. Five long COVID symptoms (abdominal pain, chest pain, sore eyes/conjunctivitis, sore throat and vomiting/nausea) were associated with reduced performance in multiple cognitive domains. Higher levels of depression and anxiety were also present in the COVID-19 group but these symptoms were mostly unrelated to cognitive performance.

**Conclusions:**

COVID-19 survivors, especially those with long COVID symptoms, are very likely to experience cognitive disruption. Measures need to be implemented to support their cognitive recovery in addition to the physical recovery.

**Disclosure:**

No significant relationships.

